# Gangrene of the Mouth in Children

**Published:** 1845-09

**Authors:** 


### SELECTIONS

FROM

### AMERICAN AND FOREIGN JOURNALS.

---

*Gangrene of the Mouth in Children.*—Dr. Hunt has published a very excellent paper on *gangrene of the mouth* in children, in the London Medical Gazette. The treatment he recommends is to commence with a purgative, composed of rhubarb, sulphate of potash, and one grain of calomel, whenever the soreness of the mouth is not so great as to render it difficult or impossible for the child to take it. In some cases it is advisable, or even necessary, to repeat this occasionally. The chief remedy, however, upon which he depends, is the chlorate of potass in solution, to the extent of from twenty to sixty grains, according to the age of the child, in divided doses, during the twenty-four hours. The beneficial effects, we are told, are often observed upon the second day of its use; almost always upon the third. The disagreeable fœtor soon diminishes; the ulcers assume a healthy, reparative action; the dribbling of saliva diminishes; and if there be simply ulceration, this very speedily heals; and if there be an eschar, it soon separates, and the ulcer granulates kindly.

We have the testimony of Mr. Cæsar Hawkins that the same treatment succeeded equally well in a case which fell under his notice. Mr. Ridson Bennett has likewise derived the most decided benefit from the internal use of the chlorate of potass in gangrene of the mouth.

In the journal last referred to, there is a paper by Robert Dunn, Esq., in relation to a fatal case of gangrene of the mouth, supervening upon measles, in a child two years and three months old, which became the subject of a coroner's inquest, in consequence of an accusation that the patient had been destroyed by the undue administration of calomel. On

6

the same day that the inquest in this case was held, Mr. Dunn was called to another case of the metastastic variety of gangrene of the mouth, in a boy four years old, also supervening upon measles. The attack came on without any premonitory indications. A small tumor formed on the inner surface of the cheek, which soon assumed a gangrenous appearance, and the breath became extremely fœtid. Externally, the surface of the affected cheek became tumid, reddish, and shining. A lotion of chloride of soda was assiduously applied, and quinia and liquor sarsæ freely given. The slough separated. The opposite cheek became in the same way affected, and the child, under the use of quinia, sarsæ, and a generous diet, was gradually progressing to convalescence at the date of Mr. Dunn's communication.—*Transac. Coll. Phys. Phila.*

